# Long‐Term Outcomes of Exercise‐Based Rehabilitation and Immunometabolic Parameters in Adult Haematopoietic Stem Cell Transplant Recipients: An Updated Systematic Review of Randomised Controlled Trials

**DOI:** 10.1002/jha2.70420

**Published:** 2026-10-03

**Authors:** Derek Dale Bayquen, Prahalad Narasimhan

**Affiliations:** ^1^ Faculty of Health and Wellbeing University of Winchester Winchester UK; ^2^ The Royal Marsden NHS Foundation Trust London UK

**Keywords:** exercise, haematopoietic stem cell transplantation, immune reconstitution, physical function, quality of life, rehabilitation, systematic review

## Abstract

**Background:**

Adult haematopoietic stem cell transplantation (HSCT) survivors have deficits in physical function, fatigue and quality of life that differ between autologous and allogeneic recipients. Few randomised controlled trials (RCTs) have evaluated exercise at 3 months or more, and immune reconstitution is rarely assessed. We synthesised RCT evidence on longer‐term exercise effects across physical, psychosocial and immunometabolic outcomes by transplant type.

**Methods:**

Following PRISMA 2020, six databases were searched (2019–2026) for RCTs comparing structured exercise with usual care or control in adults after HSCT, with outcomes at 3 months or more. Screening, extraction and risk‐of‐bias appraisal (Cochrane RoB 2) were conducted independently by two reviewers. Heterogeneity precluded meta‐analysis; findings were synthesised narratively with GRADE certainty ratings.

**Results:**

Seven RCTs (529 participants; two allogeneic, two autologous and three mixed) reported no serious exercise‐related adverse events. Between‐group 6‐min walk distance improved by approximately 51–63 m (significant in the largest trial) and sit‐to‐stand by 2.5–3.8 repetitions. Cardiorespiratory fitness, fatigue and quality‐of‐life effects were inconsistent, often within‐group only. Benefit appeared larger in allogeneic recipients, whereas an autologous trial showed none. Immunometabolic data from two trials showed an exploratory reduction in soluble ICAM‐1 and within‐group recovery of body cell mass. Certainty was low to very low.

**Conclusions:**

Exercise appears safe after HSCT and may improve objective physical function, with the greatest benefits observed in allogeneic and more impaired recipients. Cardiorespiratory, fatigue and quality‐of‐life effects remain uncertain, and immunometabolic evidence is preliminary. Future trials should report outcomes by transplant type and incorporate immune and metabolic endpoints with longer follow‐ups.

**Trial registration:**

The authors confirm that clinical trial registration is not needed for this submission

## 1 Introduction

Haematopoietic stem cell transplantation (HSCT) is a potentially curative therapy for haematological malignancies and other severe disorders, but survivors face a substantial burden of long‐term morbidity [1, 2]. Approximately two‐thirds of long‐term survivors report at least one chronic physical health condition, such as endocrine, cardiopulmonary or musculoskeletal problems [1, 2]; psychological distress is also common [2, 3]. Fatigue can persist for years after transplant [3, 4], and long‐term survivors report poorer physical, psychological and social functioning than healthy peers [4]. Survivors are also at elevated risk of cardiovascular disease and its risk factors, including hypertension, diabetes and dyslipidaemia, and of second malignancies [2, 5].

The late‐effect burden differs between transplant types, and this distinction affects both rehabilitation needs and the likely response to exercise. After autologous HSCT, most patients are not maintained on immunosuppression once engraftment is established, and relapse is the principal long‐term concern. Allogeneic recipients, by contrast, often remain on immunosuppressive therapy for extended periods and may develop chronic graft‐versus‐host disease (GVHD), corticosteroid‐related bone disease, late infections such as cytomegalovirus and iron overload [1, 2]. These ongoing exposures plausibly increase both the deconditioning that exercise might reverse and the constraints on what an intervention can achieve. Pooling the two populations therefore risks obscuring effects that are specific to one group.

Exercise‐based rehabilitation has been proposed to address these multifactorial impairments [6]. Early controlled trials suggested that supervised exercise during or soon after transplant preserves or improves strength and endurance [7, 8], with non‐significant trends favouring the intervention for quality of life (QoL) [7], and consensus panels advocate regular physical activity for cancer survivors [9]. Previous systematic reviews focused predominantly on short‐term outcomes around the transplant period [10, 11, 12, 13] and did not evaluate effects beyond the immediate recovery phase or examine emerging immunometabolic endpoints.

The objective of this review was to synthesise RCT evidence published between January 2019 and February 2026 on the effects of structured exercise‐based rehabilitation, compared with usual care or control, on physical, psychosocial, immunological and metabolic outcomes assessed at 3 months or more in adult HSCT recipients. A secondary objective was to examine whether effects differed between autologous and allogeneic recipients.

## 2 Methods

This systematic review followed the PRISMA 2020 statement [14] and was registered with the International Platform of Registered Systematic Review and Meta‐Analysis Protocols (INPLASY; registration number INPLASY202620054) on 17 February 2026; the protocol was finalised before data extraction in January 2025. The review question used the PICO structure: in adult recipients of allogeneic or autologous HSCT (Population), does participation in structured exercise‐based rehabilitation (Intervention), compared with usual care or control interventions (Comparator), improve physical, psychosocial, immunological or metabolic outcomes assessed at 3 months or more (Outcomes)? Long‐term outcomes were defined a priori as those assessed 3 months or more post‐transplant or post‐intervention.

### 2.1 Eligibility Criteria

Eligible studies were peer‐reviewed RCTs (including cluster‐RCTs) published between 1 January 2019 and 15 February 2026 that enrolled adults aged 17 years or older undergoing autologous or allogeneic HSCT for any indication. The lower age limit of 17 years was adopted to match the inclusion threshold of an eligible trial that enrolled from this age [15]. Studies conducted during hospitalisation or in the post‐transplant period were eligible provided outcomes were assessed at 3 months or more post‐transplant or post‐intervention. Interventions comprised structured exercise‐based rehabilitation incorporating aerobic, resistance, combined, interval, flexibility, balance or multimodal training in any setting and at any intensity. Co‐interventions such as dietary counselling, psychosocial support or mindfulness were permitted provided exercise was the primary component. Comparators included usual care, standard rehabilitation, attention control, waitlist or placebo/stretching. Outcomes of interest were physical function and performance (VO_2_peak, 6‐min walk distance [6MWD], grip strength, sit‐to‐stand, gait speed, Short Physical Performance Battery), fatigue, QoL, immune reconstitution, metabolic outcomes and adverse events. Non‐exercise interventions, non‐RCT designs and exclusively paediatric studies were excluded. Where multiple publications arose from a single RCT, the primary outcomes paper was prioritised.

### 2.2 Search Strategy

A comprehensive literature search was designed and executed across MEDLINE/PubMed, Embase, Scopus, PEDro (Physiotherapy Evidence Database), Web of Science, the Cochrane Central Register of Controlled Trials (CENTRAL) and the OneSearch discovery service. Each search combined haematopoietic transplantation (population) terms with exercise‐based rehabilitation (intervention) terms; study design, the comparator, outcome domains and the long‐term outcome timing criterion were applied at screening and full‐text assessment rather than within the database query, consistent with a sensitive search. Database‐specific adaptations were made for indexing conventions and controlled vocabulary. Filters for human studies and publication dates (January 2019–15 February 2026) were applied within each search without geographic restriction; the English‐language restriction was applied at screening. Reference lists of relevant systematic reviews and included studies were also screened. Initial searches were conducted in the first quarter of 2025 and updated with a confirmatory re‐run on 15 February 2026. Full database‐specific search strategies for all sources are provided in Appendix S1 in the Supporting Information.

### 2.3 Study Selection and Data Extraction

Search results were imported into reference management software and deduplicated. Titles and abstracts were screened independently by two reviewers (D.B. and P.N.) against the predefined eligibility criteria, followed by independent full‐text assessment of potentially relevant records. Disagreements at either stage were resolved by consensus after re‐examination of the source material; no adjudication by a third party was required. Standardised extraction templates were used to capture study characteristics (authors, year, country, design and sample size), population details (diagnosis, HSCT type, demographics and enrolment timing), intervention specifics (exercise type, supervision, setting, frequency, intensity and duration), control conditions and outcome data at the longest available follow‐up (mean differences, effect sizes, 95% confidence intervals and *p*‐values). Data extraction was performed in duplicate, with discrepancies reconciled through discussion. The PRISMA 2020 flow diagram summarises screening decisions (Figure 1).

**FIGURE 1:**
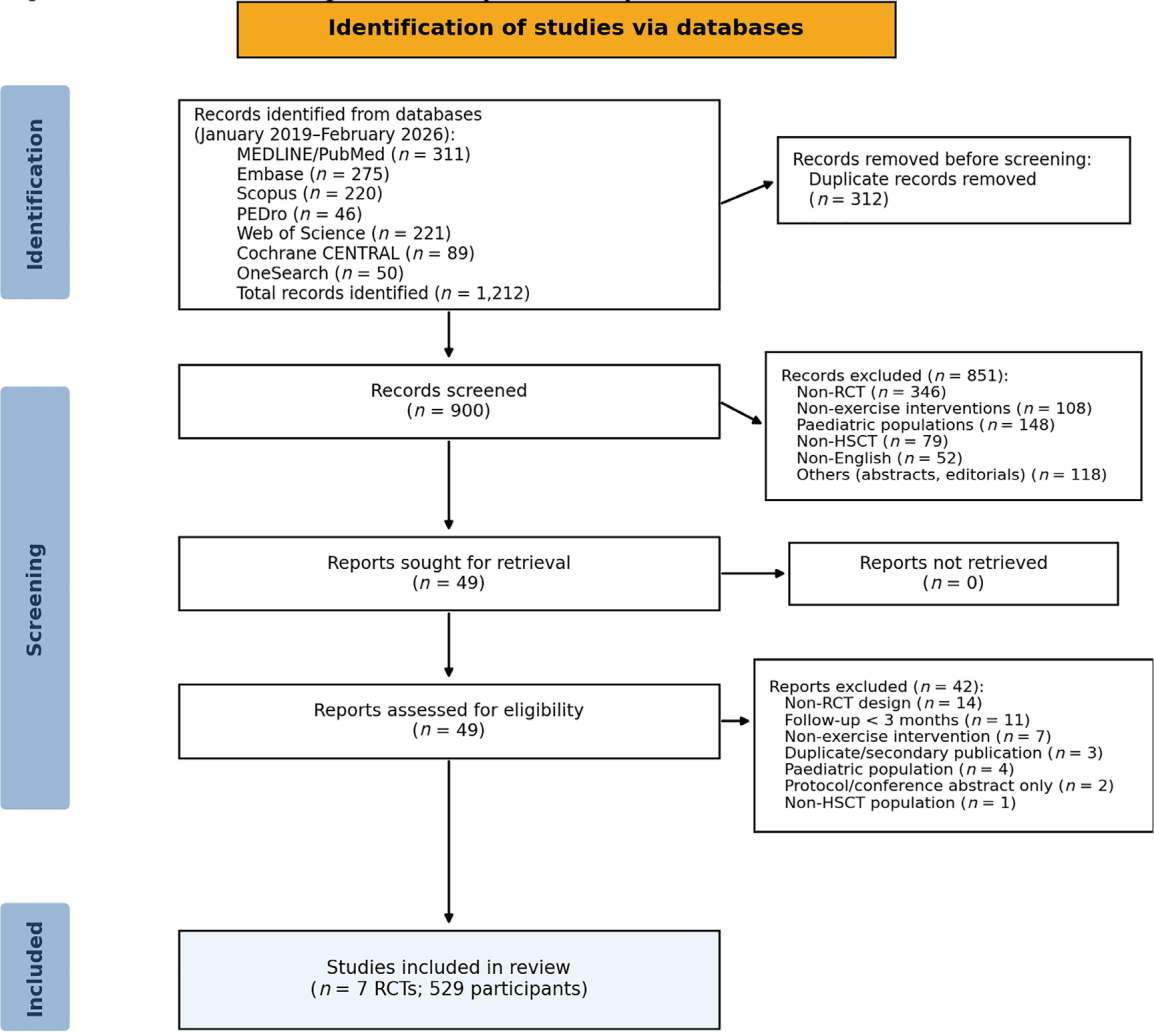
PRISMA 2020 flow diagram of study selection. Records identified across the six bibliographic databases (MEDLINE/PubMed, Embase, Scopus, PEDro, Web of Science and CENTRAL) and the OneSearch discovery service (*n* = 1212; January 2019–February 2026), duplicate records removed before screening (*n* = 312), records screened (*n* = 900), full‐text reports assessed for eligibility (*n* = 49), and randomised controlled trials included in the review (*n* = 7; 529 participants), with reasons for exclusion at each stage.

### 2.4 Risk of Bias Assessment

Included trials were appraised using the Cochrane Risk of Bias 2.0 tool (RoB 2) [16], which evaluates five domains: randomisation process (D1), deviations from intended interventions (D2), missing outcome data (D3), measurement of the outcome (D4) and selection of the reported result (D5). Each domain was rated as low risk, having some concerns or as high risk, with an overall judgement assigned per the RoB 2 algorithm: high risk if any single domain was high risk, some concerns if at least one domain raised concerns without a high‐risk rating, and low risk only if all domains were low risk. Risk of bias was appraised independently by both reviewers (D.B. and P.N.); scores were compared, and any discrepancies were resolved through discussion and reference to the supporting signalling questions. At the second revision, D.B. re‐checked every entry against the available trial reports, protocols and registry records, and P.N. reviewed each change. Two signalling questions per domain (e.g., for missing outcome data: proportion of missing data and evidence of informative missingness) were particularly influential in assigning domain‐level risk, consistent with Cochrane RoB 2.0 guidance [16]. Results are summarised in Table 2 and presented as a traffic‐light plot and a summary bar chart in Figure 2.

**FIGURE 2:**
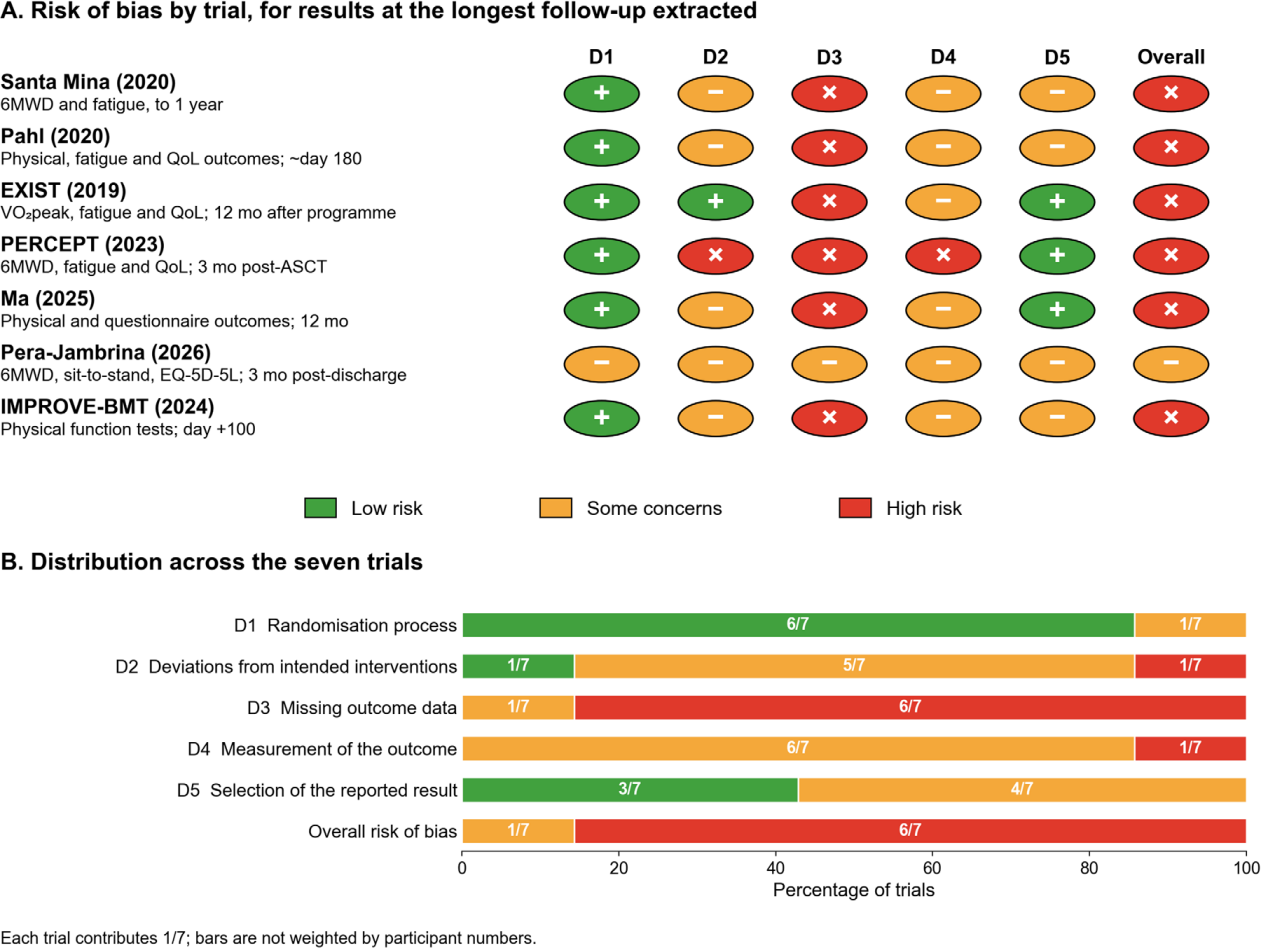
Risk‐of‐bias assessment of the included trials using the Cochrane Risk of Bias 2 tool (RoB 2). (A) Domain‐level and overall judgements for each trial, applied to its results at the longest follow‐up extracted; the outcomes and time point are given under each trial. (B) Distribution of judgements across the seven trials; each trial contributes one‐seventh, and the bars are not weighted by participant numbers. Domains: D1, randomisation process; D2, deviations from intended interventions; D3, missing outcome data; D4, measurement of the outcome; D5, selection of the reported result. Green denotes low risk, amber some concerns and red high risk.

### 2.5 Data Synthesis and Certainty of Evidence

Quantitative meta‐analysis was not performed owing to substantial clinical and methodological heterogeneity, including differences in intervention modality, timing relative to HSCT, transplant type, outcome measures and survivorship stage. Findings are presented narratively by outcome domain and, where data allowed, separately for autologous and allogeneic recipients. Clinical relevance was interpreted against published minimal important difference (MID) estimates, applied with caution because these thresholds are derived largely from other populations and have not been established for HSCT survivors (see Section 4). Pre‐specified subgroup considerations (transplant type, intervention timing, exercise modality and baseline functional status) were examined narratively given the small number of trials. Certainty of evidence for each outcome domain was rated using the GRADE approach (Table 3). Publication bias was assessed qualitatively through trial registry searches (ClinicalTrials.gov, ISRCTN and ANZCTR); no unpublished large‐scale trials were identified, precluding formal funnel‐plot analysis.

## 3 Results

### 3.1 Study Selection and Characteristics

The search identified 1212 records (January 2019 to February 2026); after removal of 312 duplicates, 900 records were screened, 49 full‐text reports were assessed for eligibility, and seven RCTs met all inclusion criteria (Figure 1). The included trials (Table 1) enrolled 529 participants across seven countries and inpatient, outpatient, home‐based and telehealth settings.

**TABLE 1:** Characteristics of included studies (n = 7).

Study (Ref)	Country	Design/setting	HSCT type	N (IG/CG)	Age (y)	Intervention	Control	Outcomes	Follow‐up
Santa Mina et al. [15]	Canada	Single‐centre feasibility RCT; inpatient → outpatient	Allogeneic	30 (15/15)	mean ∼49–50	Multiphase prehabilitation → inpatient → rehabilitation; aerobic + resistance	Usual care; home exercise programme offered after the Day‐100 assessment	6MWD, FACT‐F, MFI; VO_2_peak measured (no analysable data)	Day 100; 1 y
Pahl et al. [17]	Germany	Single‐centre RCT; inpatient	Allogeneic	71 (PP 44)	median 55–56 (PP subset)	Supervised whole‐body vibration, ∼20 min, 5×/wk during the transplant admission	Active control: mobilisation of the spine and whole‐body stretching, same schedule	VO_2_peak, chair‐rising power, body composition, MFI, QLQ‐C30	Day ±180
van Dongen et al./EXIST [18]	Netherlands	Multicentre RCT; outpatient	Autologous	109 (54/55)	mean 52	18‐wk supervised combined resistance + interval; 2×/wk (wks 1–12), 1×/wk (wks 12–18); plus 6 physical activity counselling sessions	Usual care	VO_2_peak, grip, MFI, QALYs	T1 (∼22 weeks); 12 mo after T1
McCourt et al./PERCEPT [19]	United Kingdom	Single‐centre pilot RCT; partly supervised + telehealth	Autologous (myeloma)	50 (23/27)	mean 60	Aerobic + resistance + behaviour change before/during/3 mo post‐ASCT (virtual adaptation)	Usual care	FACIT‐F, FACT‐G/BMT, 6MWT, sit‐to‐stand, IPAQ (after the COVID‐19 switch to virtual delivery, 6MWT stopped and follow‐up sit‐to‐stand self‐assessed)	3 mo post‐ASCT
Ma et al. [20]	Australia	Multicentre RCT; telehealth	Mixed (75 allo / 64 auto)	139 (73/66)	median 58	6‐wk telehealth exercise + mindfulness; 3 monthly motivation sessions (Months 3‐6)	Usual care	6MWD, sit‐to‐stand, grip, QoL, fatigue, HADS, 27 cytokines (sICAM‐1)	3, 6, 12 mo
Pera‐Jambrina et al. [21]	Spain	Single‐centre RCT; telehealth	Mixed (28 allo / 28 auto)	56 (28/28)	median 56.5	Progressive walking + squats; pedometer + weekly WhatsApp physiotherapist 3 mo post‐discharge	Same PA advice and nurse telephone number as IG; no weekly monitoring	6MWD, 30CST, EQ‐5 D‐5L	Discharge; 3 mo post‐discharge
Potiaumpai et al./IMPROVE‐BMT [22]	United States	Single‐centre pilot RCT; home‐based prehabilitation	Mixed (∼37 auto / 37 allo)	74 (36/38)	mean 60	Home‐based resistance + bodyweight prehabilitation (∼6 week pre‐HSCT)	Usual care + education	6MWT, SPPB, 30CST, TUG, grip	Pre‐HSCT; admission; +30 d; +100 d

*Note*: Lee et al. (2023; BMC Cancer) was considered but excluded because its only efficacy assessment was at 8 weeks (end of an 8‐week intervention), which does not meet the threshold of outcomes at 3 months or more post‐intervention. Total *N* = 529.

Abbreviations: 30CST, 30‐s chair‐stand test; 6MWD/6MWT, six‐min walk distance/test; ASCT, autologous stem cell transplant; CG, control group; EQ‐5 D‐5L, EuroQol 5‐dimension 5‐level; FACIT‐F/FACT‐F, Functional Assessment of Chronic Illness Therapy–Fatigue; FACT‐BMT/G, Functional Assessment of Cancer Therapy–Bone Marrow Transplant/General; HADS, Hospital Anxiety and Depression Scale; IG, intervention group; IPAQ, International Physical Activity Questionnaire; MFI, Multidimensional Fatigue Inventory; PA, physical activity; PP, per‐protocol; QALY, quality‐adjusted life year; QLQ‐C30, EORTC Quality of Life Questionnaire Core 30; RCT, randomised controlled trial; SPPB, Short Physical Performance Battery; TUG, timed up‐and‐go; VO_2_peak, peak oxygen uptake; wk, week.

Two trials enrolled exclusively allogeneic recipients (Santa Mina et al. [15], Canada; Pahl et al. [17], Germany), two exclusively autologous recipients (van Dongen et al./EXIST [18], Netherlands; McCourt et al./PERCEPT [19], United Kingdom), and three enrolled mixed allogeneic and autologous cohorts (Ma et al. [20], Australia; Pera‐Jambrina et al. [21], Spain; Potiaumpai et al./IMPROVE‐BMT [22], United States). Sample sizes ranged from 30 to 139. Mean or median participant age ranged from approximately 49–60 years. Diagnoses were predominantly multiple myeloma and lymphoma in the autologous trials, and acute leukaemias, myelodysplastic syndromes and high‐risk lymphomas in the allogeneic cohorts.

Heterogeneity was marked across three dimensions. First, transplant type, as detailed above. Second, intervention modality ranged from supervised inpatient whole‐body vibration [17] and multiphase prehabilitation‐to‐rehabilitation pathways [15] to supervised outpatient programmes [18], home‐based prehabilitation [22], and telehealth‐delivered programmes using pedometers, messaging platforms or video conferencing [19, 20, 21]. Third, timing relative to transplant varied: two trials began before or during the inpatient phase [15, 17], two were prehabilitation programmes initiated before transplant [19, 22], one began within weeks after discharge [21], one began after recovery from autologous transplant [18], and one enrolled long‐term survivors a median of 21 months post‐transplant [20].

### 3.2 Risk of Bias

Risk‐of‐bias assessments are detailed in Table 2 and visualised in Figure 2. Six trials described a random sequence and allocation concealment in the trial report, protocol or registry record, yielding low risk for Domain 1; Pera‐Jambrina et al. did not report how allocation was concealed (some concerns) [21]. Because exercise cannot be blinded, every trial was open‐label. Domain 2 was rated with some concerns for five trials [15, 17, 20, 21, 22] and high risk for PERCEPT, because the control group was probably contaminated [19]. EXIST was rated low risk: usual care left control participants free to be physically active and to use healthcare services [18], so any exercise or physiotherapy they took up counted as part of the comparator. Missing outcome data (Domain 3) produced high risk in six trials: Santa Mina et al. retained approximately one‐third of participants by 1 year [15], Pahl et al. analysed 44 of 71 randomised patients who completed at least two of three assessments [17], and in four trials losses were large or differed between arms, were often for health‐related reasons, and were handled by analyses assuming data were missing at random without sensitivity analyses [18, 19, 20, 22]. Pera‐Jambrina et al. had physical test data for most participants at 3 months (some concerns) [21]. No trial reported blinded outcome assessment (Domain 4), although objective performance tests partially mitigated measurement bias; PERCEPT was rated high risk because participants who knew their allocation and preferred the exercise arm self‐reported fatigue and QoL [19]. For Domain 5, three trials were rated low risk [18, 19, 20] and four had some concerns, because no analysis plan could be shown to predate the outcome data [15, 17, 21, 22]; six trials were registered, and Pera‐Jambrina et al. reported no registration [21]. No trial achieved an overall low risk‐of‐bias judgement. Six trials were rated as high risk overall, mainly because of missing outcome data [15, 17, 18, 19, 20, 22], and Pera‐Jambrina et al. was rated with some concerns [21].

**TABLE 2:** Risk of bias assessment (Cochrane RoB 2).

Study	D1 randomisation	D2 deviations	D3 missing data	D4 measurement	D5 reporting	Overall
Santa Mina et al. [15]	Low	Some concerns	High	Some concerns	Some concerns	High
Pahl et al. [17]	Low	Some concerns	High	Some concerns	Some concerns	High
van Dongen et al./EXIST [18]	Low	Low	High	Some concerns	Low	High
McCourt et al./PERCEPT [19]	Low	High	High	High	Low	High
Ma et al. [20]	Low	Some concerns	High	Some concerns	Low	High
Pera‐Jambrina et al. [21]	Some concerns	Some concerns	Some concerns	Some concerns	Some concerns	Some concerns
Potiaumpai et al./IMPROVE‐BMT [22]	Low	Some concerns	High	Some concerns	Some concerns	High

*Note*: Domain rationale: D1 low for six (random sequence; concealment described in the trial report, protocol or registry record); some concerns for Pera‐Jambrina et al. (allocation concealment not reported). D2 reflects open‐label design inherent to exercise: low for EXIST (control‐group physiotherapy permitted by the protocol and part of usual care); some concerns for five; high for PERCEPT (probable contamination of the control group only). D3 high for six (large or unequal losses to follow‐up, often for health‐related reasons, with no sensitivity analysis; Santa Mina et al. ∼67% attrition by 1 year; Pahl et al. analysis of the 44 of 71 with at least two of three assessments); some concerns for Pera‐Jambrina et al. D4 some concerns for six (no blinded outcome assessment; objective tests partially mitigate); high for PERCEPT (self‐reported outcomes from participants who knew their allocation and preferred the exercise arm). D5 low for EXIST and PERCEPT (analyses followed the protocol or departed from it for stated reasons) and for Ma et al. (the protocol predates enrolment and the statistician's outcome analyses were blinded; the reported model departs from the protocol without a stated reason); some concerns for the other four (no analysis plan shown to predate the outcome data; Pera‐Jambrina et al. reports no registration). Judgements apply to the result at the longest follow‐up extracted. Overall: six high, one with some concerns (Pera‐Jambrina et al.); none low.

### 3.3 Physical Function

#### 3.3.1 Aerobic Capacity and Walking Performance

The 6MWD was the most consistently reported measure. Ma et al. [20] reported a between‐group improvement of 51.4 m at 3 months (95% CI 27.4–75.5; *p* < 0.001; effect size 0.52) that was sustained at 59.3 m at 12 months (95% CI 20.4–98.1; *p* = 0.003; effect size 0.60), with a significant group‐by‐time interaction (*p* = 0.001). In the IMPROVE‐BMT prehabilitation trial, the exercise group walked 62.8 m further than usual care group at 100 days post‐transplant, though this between‐group difference did not reach significance (95% CI−10.3 to 135.9; *p* = 0.09); at Day 100, relative to the study baseline before prehabilitation, mean distance was 25 m higher in the exercise group and 12 m lower in usual care, although neither within‐group change was significant [22]. Pera‐Jambrina et al. [21] found no significant between‐group difference in absolute 6MWD at 3 months (mean 484.8 vs. 441.1 m; *p* = 0.18); however, in within‐group analysis the intervention group recovered to its pre‐admission distance while the control group remained 41 m below baseline (*p* = 0.048), an effect that was more pronounced in the allogeneic subgroup, where the between‐group difference reached 77 m. Santa Mina et al. [15] reported a within‐group gain of 45 m from baseline to pre‐transplant in the exercise group but did not perform between‐group significance testing owing to sample‐size constraints, and walking distance declined in both groups between the pre‐transplant and 100‐day assessments. In the EXIST trial, the programme had no significant effect on cardiorespiratory fitness directly after the intervention, and there was no between‐group difference 12 months after it ended (0.12 mL·kg^−1^·min^−1^; 95% CI −1.89 to 2.14; *p* = 0.90) [18]. PERCEPT reported descriptive trends toward greater improvement in the exercise group during the prehabilitation phase, without formal significance testing [19].

Direct VO_2_peak data were limited. Pahl et al. [17] reported a within‐group increase in relative VO_2_peak in the intervention group at the Day‐180 follow‐up (*p* = 0.035), with no corresponding increase in the control group, whereas EXIST found no between‐group difference directly after the programme or 12 months later [18]. Across trials, the evidence for a durable cardiorespiratory fitness benefit was therefore inconsistent.

#### 3.3.2 Lower‐Body Strength

Sit‐to‐stand or chair‐stand performance improved consistently where measured. Ma et al. [20] reported between‐group differences of 2.5 repetitions per 30 s at 3 months (95% CI 1.2–3.8; *p* < 0.001) and 3.2 repetitions at 12 months (95% CI 1.6–4.8; *p* < 0.001), with a significant group‐by‐time interaction. IMPROVE‐BMT reported a between‐group difference of 3.79 thirty‐second chair stands at Day 100 (95% CI 0.74–6.84; *p* = 0.02), with the exercise group improving and usual care declining [22]. Pera‐Jambrina et al. [21] reported a within‐group gain of 1.7 repetitions in the intervention group versus a 0.8‐repetition decline in controls at 3 months, with a between‐group difference among allogeneic recipients of 3.8 repetitions, although the overall between‐group comparison did not reach significance (*p* = 0.09). Pahl et al. [17] reported improvement in chair‐rising power in the intervention group (*p* = 0.009). Grip strength showed no consistent between‐group difference in any trial.

### 3.4 Fatigue

Fatigue was assessed with several instruments, complicating comparison. Pahl et al. [17] reported a within‐group reduction in Multidimensional Fatigue Inventory total score in the intervention group with a between‐group difference at follow‐up (*p* = 0.038), alongside higher fatigue in the control group at discharge. In PERCEPT, within‐group fatigue (FACIT‐Fatigue) improved beyond the MID in the exercise group during prehabilitation (+5.7; 95% CI 0.1–11.2) but not in controls [19]. Santa Mina et al. [15] reported a within‐group improvement in fatigue‐related scales at later timepoints, but attrition precluded reliable between‐group inference. In contrast, the EXIST trial found no between‐group difference in general fatigue at 12 months (−0.69; 95% CI −2.52 to 1.14; *p* = 0.46) [18], and Ma et al. [20] found no significant between‐group difference on fatigue subscales, although 6MWD improvement correlated with trends in fatigue and mood. The pattern across trials suggested that fatigue benefit was more apparent when exercise was delivered during or close to the transplant period than in the late‐survivorship setting, although this observation is based on indirect comparison across heterogeneous instruments and should be regarded as hypothesis‐generating.

### 3.5 Quality of Life

Global QoL indices were largely unchanged, whereas physical and role‐functioning domains were more responsive. Pahl et al. [17] reported within‐group improvements in EORTC QLQ‐C30 physical functioning (*p* = 0.035) and role functioning (*p* = 0.025) in the intervention group at follow‐up, with control‐group worsening of skin side effect scales. Ma et al. [20] found no significant between‐group difference in global QoL at 3 or 12 months, but the group‐by‐time interaction for HADS anxiety was significant (*p* = 0.002). Pera‐Jambrina et al. [21] reported a significant between‐group advantage in the EQ‐5 D‐5L usual‐activities dimension at 3 months (*p* = 0.009); the self‐care dimension did not differ (*p* = 0.61). PERCEPT reported within‐group improvements beyond the MID in FACT‐G and FACT‐BMT in the exercise group during prehabilitation, without between‐group significance testing [19]. The EXIST trial found no quality‐adjusted life‐year benefit, and the intervention was not cost‐effective [18]. Physical and daily‐activity facets therefore improved modestly in several trials, whereas emotional and social domains were generally unchanged.

### 3.6 Immunometabolic Outcomes

Immunological and metabolic biomarker data were reported by only two trials. In an exploratory analysis of 27 serum cytokines, Ma et al. [20] found a significant between‐group reduction in soluble intercellular adhesion molecule‐1 (sICAM‐1) from baseline to 3 months in the intervention group (mean difference −0.23 ng·mL^−1^; 95% CI −0.42 to −0.04; *p* = 0.003), a marker of vascular inflammation and endothelial activation. No other cytokine differed significantly between groups; cytokines were measured only at baseline and after the 3‐month assessment, in 69 of 139 participants who provided optional blood samples. Body composition data were available from Pahl et al. [17] as within‐group changes: at the Day‐180 follow‐up, body cell mass (*p* = 0.002) and phase angle fell significantly in the control group, whereas the intervention group regained baseline body cell mass; fat‐free mass percentage rose in both groups, significantly only in the intervention group (*p* = 0.035). No trial measured lipid profiles, glycaemic markers, metabolic syndrome incidence, lymphocyte subset reconstitution, immunoglobulin levels or vaccination immunogenicity, precluding any inference on exercise‐mediated immune or cardiometabolic modification.

### 3.7 Safety

No serious exercise‐related adverse events were reported in any trial. In the study by Santa Mina et al. [15], one serious adverse event (a fall during a baseline 6MWD test causing subarachnoid haemorrhage) occurred in a participant who had not been randomised and was unrelated to the intervention. Whole‐body vibration was permitted at platelet counts down to 10,000/µL, and no severe adverse events such as bleeding occurred [17]. IMPROVE‐BMT reported zero exercise‐related adverse events with 92% adherence [22]. In PERCEPT, two adverse events occurred (one per arm), with one mild episode of dizziness possibly intervention‐related and no serious events [19]. One‐year survival did not differ between arms in IMPROVE‐BMT [22], and none of the included trials was powered to compare survival, infection, GVHD or relapse.

## 4 Discussion

This systematic review synthesised seven RCTs published between 2019 and early 2026 examining exercise‐based rehabilitation in adult HSCT recipients, with outcomes assessed at 3 months or more. The evidence suggests that exercise delivered across the transplant continuum appears safe and may improve objective lower‐body strength and walking performance, whereas effects on cardiorespiratory fitness, fatigue and global QoL are inconsistent. The most reproducible gains were in 6MWD, where between‐group improvements of approximately 51–63 m were reported, reaching significance in the largest trial, and in sit‐to‐stand performance, with gains of about 2.5–3.8 repetitions.

### 4.1 Interpreting Clinical Relevance

These gains should be interpreted against MID estimates with explicit caution. No MID for improvement in 6MWD has been established in haematological malignancies; a published estimate for deterioration, derived in lung cancer, is 22 to 42 m [23], and a meta‐analysis in transplant and haematology populations reported a pooled improvement of 29 m, which the authors classified against this borrowed threshold [13]. The between‐group walking gains in the present review fall at or above this range, but applying a lung‐cancer threshold for deterioration to gains in HSCT survivors is an approximation. Similarly, while a difference of two or more repetitions on chair‐stand testing is commonly regarded as meaningful, thresholds derived for the five‐repetition sit‐to‐stand test should not be transferred directly to the 30‐s chair‐stand test used in most included trials, because the two assessments have different scaling and psychometric properties. We have therefore avoided asserting definitive clinical‐significance thresholds and instead report the direction and magnitude of effects alongside the relevant caveats.

### 4.2 Autologous Versus Allogeneic Stem Cell Transplants

The benefit of exercise concentrates in particular transplant groups. In the autologous EXIST trial, fitness and fatigue did not differ between arms directly after the programme or 12 months later, and the programme was not cost‐effective; the authors suggested that exercise after transplant may not speed natural recovery [18]. By contrast, benefit appeared greatest where baseline impairment was higher. Pera‐Jambrina et al. [21] found more pronounced walking and chair‐stand recovery in the allogeneic subgroup than in autologous recipients, and the allogeneic peri‐transplant trials reported preservation of strength and fatigue relative to declining controls [15, 17], with body cell mass recovered within the exercise group in the study by Pahl et al. [17]. This pattern is biologically coherent: allogeneic recipients, who face chronic GVHD, prolonged immunosuppression, corticosteroid‐related bone disease and late infections, have more deconditioning available to reverse and a longer trajectory over which to benefit. The implication is that exercise may add most value in allogeneic and more impaired recipients, and least in uncomplicated autologous recovery. A limitation of the current evidence is that the three mixed‐cohort trials largely reported pooled outcomes rather than results stratified by transplant type, so most transplant‐specific inference rests on the two exclusively allogeneic trials and the subgroup data of a single mixed trial. Future trials should report outcomes separately for autologous and allogeneic recipients.

### 4.3 Sources of Heterogeneity

Several identifiable factors account for the variability across trials. Intervention modality ranged from whole‐body vibration and supervised gym‐based programmes to home‐based and telehealth delivery, with differing physiological targets. Timing relative to transplant spanned prehabilitation, inpatient conditioning, early discharge and late survivorship, and the trials beginning during or close to transplant tended to report larger fatigue and functional effects than those in established survivors, consistent with a larger reversible deconditioning deficit earlier in the trajectory. Patient characteristics differed in transplant type and time since transplant, with the only late‐survivor cohort showing a distinct response pattern. Follow‐up duration ranged from Day 100 to 12 months, and outcome assessment used several different fatigue and QoL instruments, which alone precluded quantitative pooling. These sources of heterogeneity were the basis for the decision to synthesise narratively rather than by meta‐analysis.

### 4.4 Outcome‐Level Synthesis

Considered by domain, the evidence is most consistent for lower‐body strength, where four trials reported gains favouring exercise, two of them significant between‐group and two within‐group, with no contradictory findings. Walking performance was directionally consistent across five trials but reached between‐group significance in only one. Cardiorespiratory fitness, fatigue and global QoL were inconsistent, with significant effects in some trials offset by null findings in the larger trials, indicating very low certainty. Immunometabolic and body‐composition signals each rested on a single trial. This graded picture, reflected in the GRADE ratings (Table 3), suggests that confidence is low for the functional‐strength and walking benefits and low to very low for the remaining outcomes.

**TABLE 3:** GRADE summary of findings.

Outcome domain	Trials (*n*)	Direction/magnitude	Certainty (GRADE)	Reasons for downgrading
Lower‐body strength (sit‐to‐stand/chair‐stand)	4 [17, 20, 21, 22]	Consistent benefit; between‐group +2.5 to +3.8 reps where significant	Low	Risk of bias (three of four trials at high risk overall, mainly from missing outcome data; no blinding), imprecision (small trials), indirectness (mixed tests)
Walking performance (6MWD)	5 [15, 19, 20, 21, 22]	Directionally favourable; +51 to +63 m, significant in 1 trial	Low	Risk of bias (four of five trials at high risk overall, mainly from missing outcome data), inconsistency (significant in one trial only), imprecision
Cardiorespiratory fitness (VO_2_peak)	2 [17, 18]	Inconsistent; within‐group gain in one, null at 12 mo in other	Very low	Risk of bias (both trials at high risk overall), inconsistency, imprecision
Fatigue	5 [15, 17, 18, 19, 20]	Mixed; benefit peri‐transplant, null in survivorship	Very low	Risk of bias (all five trials at high risk overall), inconsistency, indirectness (varied instruments)
Quality of life (global/domain)	5 [17, 18, 19, 20, 21]	Global unchanged; physical/daily‐activity domains improved in some	Very low	Risk of bias (four of five trials at high risk overall), inconsistency, imprecision
Inflammatory biomarker (sICAM‐1)	1 [20]	Reduced at 3 mo (−0.23 ng·mL^−^^1^; *p* = 0.003); no later samples	Very low	Single trial, risk of bias (trial at high risk overall; one of 27 cytokines, not named in the trial protocol; measured in 69 of 139 participants), imprecision
Body composition	1 [17]	Within‐group only: body cell mass fell significantly in controls and returned to baseline with exercise; fat‐free mass percentage rose in both arms	Very low	Single trial, risk of bias (high risk overall), imprecision
Safety (serious adverse events)	7	No serious exercise‐related events	Low	Risk of bias (six of seven trials at high risk overall); rare‐event imprecision

*Note*: GRADE certainty starts at high for RCT evidence and was downgraded for risk of bias, inconsistency, indirectness, imprecision and (where applicable) single‐study evidence. Trial counts reflect studies contributing analysable between‐group data to each outcome; some trials measured an outcome but did not report usable between‐group data (for example VO_2_peak in Santa Mina et al., not analysable because many participants did not reach maximal effort on exercise testing, and sit‐to‐stand in McCourt et al./PERCEPT, measured but not reported as a between‐group result). Risk of bias was downgraded by one level (serious) for lower‐body strength and walking performance: in the largest trial, between‐group gains in walking distance (51.4 m) and sit‐to‐stand were already present at 3 months, when 8% of assessments were missing, and were sustained at 12 months [20].

### 4.5 Immunometabolic Evidence

Evidence for immunometabolic modification is preliminary and is the least‐studied outcome domain in this review. Only one trial measured an inflammatory biomarker, reporting, in an exploratory analysis, a modest but significant reduction in sICAM‐1 at 3 months that is compatible with an anti‐inflammatory effect of exercise [20], although cytokines were measured only at baseline and after the 3‐month assessment, and no other cytokine differed between groups. Within‐group body‐composition changes were reported in one further trial [17]. No trial assessed lymphocyte subset reconstitution, T‐cell receptor diversity, vaccination immunogenicity, lipid trajectories, glycaemic control or metabolic syndrome incidence. Given the cardiometabolic and immune burden of HSCT survivorship, this is a priority for future trial design.

### 4.6 Methodological Considerations

Sequence generation was adequate in all trials, and six of the seven were registered, but no trial achieved an overall low risk of bias, reflecting the inherent impossibility of blinding exercise and the losses to follow‐up, often for health‐related reasons, seen in six of the seven trials. The two inpatient allogeneic trials reported some of the larger within‐group effects, consistent with the general pattern that studies at higher risk of bias tend to report larger effects; the more conservative between‐group estimates from larger trials such as EXIST may therefore better represent true efficacy, although EXIST was also at high risk of bias from missing outcome data. The use of multiple fatigue and QoL instruments precluded quantitative synthesis, and adoption of harmonised core outcome sets would improve future comparability.

Studies other than RCTs fell outside the eligibility criteria; those cited here were not appraised for risk of bias or synthesised and provide context for the RCT findings. In a mainly autologous cohort, a prospective controlled study that did not report how participants were allocated delivered exercise from before transplant to Day 100. At Day 100, lower‐limb strength, global QoL and the physical impact of fatigue favoured the exercise group, and walking distance did not differ between groups [24]; the QoL and fatigue findings, which the RCTs showed inconsistently, may partly reflect the unreported allocation method. In allogeneic recipients, a prospective study of sequential cohorts found higher odds of being fit at transplant admission after telemedicine‐supervised prehabilitation and no significant difference in 1‐year survival [25], and a retrospective administrative database study associated pre‐transplant rehabilitation with a shorter post‐transplant hospital stay [26]. In autologous myeloma recipients, an uncontrolled service evaluation found impaired function at baseline in most patients and within‐group gains in walking, sit‐to‐stand and QoL to Day 100 [27], which, without a control group, cannot be attributed to the programme.

## 5 Limitations

This review has several limitations. The small number of trials and marked heterogeneity in modality, timing, population and instruments constrain the strength of conclusions. Follow‐up rarely exceeded 12 months, leaving longer‐term effects on morbidity, mortality and healthcare use unknown. The restriction to English‐language publications from 2019 onward, adopted to capture contemporary practice, may have excluded relevant non‐English or earlier trials. Most trials originated from high‐income countries, limiting generalisability to resource‐constrained settings. Publication bias, although assessed qualitatively, cannot be excluded, and narrative synthesis precludes pooled quantitative estimates. The protocol, although finalised before data extraction, was registered retrospectively (February 2026) rather than before the searches began.

## 6 Future Research Priorities

Trials should report outcomes separately for autologous and allogeneic recipients, employ harmonised core outcome sets, and incorporate pre‐specified immunological endpoints (lymphocyte subsets, inflammatory cytokine panels, vaccination titres) and metabolic parameters (fasting lipids, HbA1c and body composition). Adequately powered multicentre trials with follow‐up beyond 12 months are needed to evaluate effects on immune reconstitution, cardiometabolic risk and survival. Hybrid effectiveness‐implementation designs would inform real‐world integration, and pragmatic low‐technology and telehealth models merit evaluation in resource‐constrained settings.

## 7 Conclusions

This updated review of seven RCTs suggests that structured exercise‐based rehabilitation appears safe and may improve objective physical function, particularly lower‐body strength and walking performance, in adult HSCT recipients. Benefit appears greatest in allogeneic and more impaired recipients and least in uncomplicated autologous recovery, supporting transplant‐type‐specific referral and trial design. Effects on cardiorespiratory fitness, fatigue and global QoL remain uncertain, and certainty of evidence is low to very low across outcomes. Immunometabolic evidence is preliminary and is the principal evidence gap. These findings support offering exercise in HSCT survivorship care. Adequately powered trials that report by transplant type and incorporate immune, metabolic and long‐term health endpoints are needed.

## Author Contributions

**Dale Bayquen**: conceptualisation, methodology, investigation, data curation, formal analysis, risk‐of‐bias assessment, writing – original draft, writing – review and editing, project administration. **Prahalad Narasimhan**: conceptualisation, methodology, investigation, data curation, risk‐of‐bias assessment, supervision, writing – review and editing.

## Funding

The authors have nothing to report.

## Ethics Statement

This study did not require ethical approval as it is a systematic review based exclusively on previously published, peer‐reviewed literature, with no primary collection or analysis of patient data, identifiable information, or biological samples. The work was undertaken with academic and institutional support from the University of Winchester, and all included studies were assumed to have obtained their own ethical approvals as reported in their respective publications.

## Conflicts of Interest

The authors declare no conflicts of interest.

## Supporting information




**Supporting File**: jha270420‐sup‐0001‐Appendix.docx

## Data Availability

All data analysed in this review are derived from the published articles cited in the reference list. No new primary data were generated.
