# Supplementary material for: Long‐Term Outcomes of Exercise‐Based Rehabilitation and Immunometabolic Parameters in Adult Haematopoietic Stem Cell Transplant Recipients: An Updated Systematic Review of Randomised Controlled Trials
Source: EJHaem. 2026 Oct 3;7(5):e70420. doi: 10.1002/jha2.70420 (PMC13633763; doi:10.1002/jha2.70420)
Supplement: Supplementary file 1 [file JHA2-7-e70420-s001.docx]

**Supplementary Appendix S1. Database-specific search strategies**

The database searches used a sensitive two-concept structure combining the population (haematopoietic stem cell transplantation) with the intervention (exercise-based rehabilitation). Consistent with the registered eligibility criteria (INPLASY202620054) and with standard practice for sensitive searching, study design (randomised controlled trials), the comparator, the outcome domains and the three-month outcome-timing criterion, and the English-language restriction, were applied at the title and abstract screening and full-text eligibility stages rather than within the database query. This maximises sensitivity at identification and accounts for the records removed during screening, including non-randomised designs, non-exercise interventions and non-English reports (Figure 1). A human-studies filter and the date limits (1 January 2019 to 15 February 2026) were applied within each search. Initial searches were run in the first quarter of 2025 and updated with a confirmatory re-run on 15 February 2026. Exact retrieval counts per source are reported in the PRISMA 2020 flow diagram (Figure 1).

Records were screened against the eligibility criteria independently by two reviewers (DB and PN), with disagreements resolved by consensus. In addition to the database searches below, the reference lists of relevant systematic reviews and included studies were also screened.

**S1.1 MEDLINE (PubMed)**

Concept 1 (population): "Hematopoietic Stem Cell Transplantation"[Mesh] OR "Bone Marrow Transplantation"[Mesh] OR hematopoietic stem cell transplant*[tiab] OR haematopoietic stem cell transplant*[tiab] OR bone marrow transplant*[tiab] OR HSCT[tiab]

Concept 2 (intervention): "Exercise"[Mesh] OR "Exercise Therapy"[Mesh] OR "Resistance Training"[Mesh] OR exercise[tiab] OR "physical activity"[tiab] OR rehabilitation[tiab] OR "resistance training"[tiab] OR "aerobic training"[tiab] OR prehabilitation[tiab]

Executed: (Concept 1) AND (Concept 2). Filters: Humans; 2019/01/01:2026/02/15.

**S1.2 Embase (Elsevier; Emtree)**

Concept 1 (population): 'hematopoietic stem cell transplantation'/exp OR 'bone marrow transplantation'/exp OR (haematopoietic NEXT/1 stem NEXT/1 cell NEXT/1 transplant*):ab,ti OR hsct:ab,ti

Concept 2 (intervention): 'exercise'/exp OR 'kinesiotherapy'/exp OR 'resistance training'/exp OR exercise:ab,ti OR 'physical activity':ab,ti OR rehabilitation:ab,ti OR 'aerobic training':ab,ti OR prehabilitation:ab,ti

Executed: #1 AND #2. Limits: [humans]/lim AND [2019-2026]/py.

**S1.3 Scopus (TITLE-ABS-KEY)**

( TITLE-ABS-KEY ( "hematopoietic stem cell transplant*" OR "bone marrow transplant*" OR HSCT ) ) AND ( TITLE-ABS-KEY ( exercise OR "physical activity" OR rehabilitation OR "resistance training" OR "aerobic training" OR prehabilitation ) ) AND ( PUBYEAR > 2018 AND PUBYEAR < 2027 )

**S1.4 PEDro (Physiotherapy Evidence Database)**

Abstract & Title: exercise transplantation. Published since 2019; published before 2026. PEDro uses a field-based search, so the population and intervention concepts were entered in the abstract/title field; PEDro indexes controlled trials by design.

**S1.5 Web of Science (Core Collection; Topic = TS)**

TS=("hematopoietic stem cell transplant*" OR "bone marrow transplant*" OR HSCT) AND TS=(exercise OR "physical activity" OR rehabilitation OR "resistance training" OR "aerobic training" OR prehabilitation). Publication years 2019–2026; document type Article.

**S1.6 Cochrane CENTRAL (CRSO)**

#1 MeSH descriptor [Hematopoietic Stem Cell Transplantation] explode all trees; #2 MeSH descriptor [Bone Marrow Transplantation] explode all trees; #3 (haematopoietic stem cell transplant* OR bone marrow transplant* OR HSCT):ti,ab,kw; #4 #1 OR #2 OR #3; #5 MeSH descriptor [Exercise] explode all trees; #6 (exercise OR physical activity OR rehabilitation OR resistance training OR aerobic training OR prehabilitation):ti,ab,kw; #7 #5 OR #6; #8 #4 AND #7 (publication year 2019 to 2026).

**S1.7 OneSearch (University of Winchester discovery service)**

The population and intervention concepts were combined with AND using the same free-text terms applied across the bibliographic databases, limited to 2019–2026 and to human studies. OneSearch was searched as a supplementary discovery layer.

Note: study design (randomised controlled trials), the comparator, the outcome domains, the three-month outcome-timing criterion and the English-language restriction were applied during title/abstract screening and full-text assessment, not within the database queries above. Exact retrieval counts per source are recorded in the PRISMA 2020 flow diagram (Figure 1).
